# Dexrazoxane Cardioprotection in pediatric ALL: a historical control cohort study

**DOI:** 10.1186/s40959-025-00420-8

**Published:** 2026-01-09

**Authors:** Li Liu, Hongping Yang, Yan Zhou, Na Li, Qi Nie, Xinmiao Liu, Chunhui Yang, Yue Tian, Xiaoyan Mao, HaiJin Li, Qulian Guo, Xin Tian

**Affiliations:** 1 Institute of Advanced Biotechnology, Institute of Homeostatic Medicine, and School of Medicine, Southern University of Science and Technology, 518055 Shenzhen, China; 2 Department of Pediatrics, Qujing Medical College, Qujing, 655100 China; 3 Department of Hematology, Kunming Children’s Hospital, 650118 Kunming, China; 4 Department of Pediatrics, Dali University, Dali, 671003 China; 5 Department of Pediatrics, Sichuan Clinical Research Center for Birth Defects, The Affiliated Hospital of Southwest Medical University, Luzhou, 646000 China; 6 Department of Pediatrics, Sichuan Provincial Woman’s and Children’s Hospital / The Affiliated Women’s and Children’s Hospital of Chengdu Medical College, Chengdu, 610000 China

**Keywords:** Pediatric acute lymphoblastic leukemia, Anthracyclines, Cardiotoxicity, Cardioprotective strategy, Safety

## Abstract

**Objective:**

This ambispective cohort study evaluates the effectiveness of cardioprotective strategies in reducing anthracycline-induced cardiotoxicity in pediatric patients with acute lymphoblastic leukemia (ALL).

**Methods:**

We conducted a single-center, historically controlled ambispective cohort study at Kunming Children’s Hospital. Children with newly diagnosed ALL treated with the CCLG-ALL2018 protocol between May 2018 and May 2020 comprised the retrospective historical control cohort (no dexrazoxane). After institutional adoption of dexrazoxane, consecutive eligible patients treated between May 2020 and May 2022 were prospectively enrolled and followed according to a prespecified cardiac monitoring schedule. The exposure (use of dexrazoxane) was determined by calendar time rather than randomization.

**Results:**

The combination therapy group exhibited significantly lower rates of electrocardiogram and echocardiogram abnormalities (*P* < 0.01 and *P* < 0.01, respectively), suggesting a notable cardioprotective effect. Although baseline cardiac markers did not show significant differences between the groups, post-treatment levels of cardiac troponin T were significantly improved in the combination therapy group (*P* < 0.05). Additionally, trends toward improved left ventricular ejection fraction and Creatine Kinase-Myocardial Band levels were observed, although these did not reach statistical significance. Importantly, the use of the cardioprotective agent did not lead to a significant increase in adverse reactions.

**Conclusion:**

Incorporating a cardioprotective strategy into pediatric ALL treatment protocols appears to lower the risk of anthracycline-induced cardiotoxicity without compromising safety, hence boosting patient outcomes. These findings should be interpreted in light of the historically controlled, nonrandomized design and potential time-related confounding.

## Introduction

 Anthracyclines play a crucial role in the treatment of a broad spectrum of solid tumors and hematological malignancies due to their potent antineoplastic properties. Approximately 50% of pediatric cancer patients, including those diagnosed with acute lymphoblastic leukemia (ALL), acute myeloid leukemia (AML), and different lymphomas, require anthracycline-based chemotherapy during their treatment [1, 2]. While anthracyclines have shown substantial efficiency in fighting these malignancies, their clinical application is clouded by the production of various adverse effects, most notably cardiotoxicity. Studies have revealed that roughly 10% of pediatric patients treated with anthracyclines may develop heart failure, accompanied by a period of clinical symptoms [3, 4]. This significant risk of cardiotoxicity imposes constraints on the use of anthracyclines.

Addressing the difficulty of minimizing cardiotoxic effects generated by anthracycline therapy has arisen as a critical medical dilemma. In recent years, numerous cardioprotective strategies have received interest for their possible involvement in preventing cardiomyocyte death and increasing cardiac performance. Evidence increasingly supports the cardioprotective features of these strategies, suggesting their efficacy in ameliorating heart harm produced by anthracycline treatment [5–7]. Additionally, several worldwide investigations have validated the preventive effects of these strategies against anthracycline-induced cardiac damage [8, 9]. However, in China, there is a substantial shortage of high-quality clinical research addressing cardioprotective strategies to prevent cardiotoxicity generated by anthracyclines in pediatric patients with acute lymphoblastic leukemia.

This study aims to bridge this gap through an ambispective cohort study with historical controls that investigates the clinical value of incorporating cardioprotective strategies into treatment regimens for pediatric ALL patients undergoing anthracycline therapy. By focusing on this under-researched area, the study endeavors to provide substantial evidence supporting the integration of cardioprotective strategies into therapeutic regimens, potentially revolutionizing the approach to safeguarding cardiac health in pediatric oncology.

## Materials and methods

### Study design and participants

This ambispective cohort study with historical controls analyzed 260 pediatric patients with acute lymphoblastic leukemia treated at our institution between May 2018 and May 2022. The historical control group consisted of 125 patients (61 males, 64 females; mean age 6.21 ± 1.12 years) who received anthracycline-based chemotherapy alone from May 2018 to May 2020, with data retrospectively extracted from medical records. The intervention group comprised 135 patients (68 males, 67 females; mean age 6.35 ± 1.08 years) who received anthracycline combined with dexrazoxane from May 2020 to May 2022 and were prospectively monitored according to a predetermined protocol. The addition of dexrazoxane to the standard CCLG-ALL2018 protocol was implemented as an institutional practice change in May 2020 based on emerging evidence of its cardioprotective benefits.

Baseline characteristics including age, gender, ALL risk stratification, immunophenotype, and initial laboratory values were compared between groups, showing no significant differences (all *P* > 0.05), ensuring comparability. The study protocol was approved by the hospital’s Ethics Committee (2022-03-049-K01). A waiver of informed consent was granted for retrospective data collection from the historical control group, while informed consent was obtained from patients and guardians in the prospective intervention group.

Inclusion and Exclusion Criteria.

(1)Inclusion Criteria:

For both groups: Patients diagnosed with ALL; anthracycline chemotherapy dosages below the known threshold for cardiotoxicity (cumulative dose < 300 mg/m²).

For the historical control group: Complete medical records available for review including baseline characteristics, treatment details, and cardiac monitoring results.

For the prospective intervention group: Patients and their families willing to participate and able to understand the study objectives with written informed consent obtained.

(2) Exclusion Criteria: Pre-existing contraindications to chemotherapy; abnormal myocardial enzyme levels or cardiac abnormalities detected by color Doppler echocardiography; significant organ damage other than the heart; severe neurological or psychiatric diseases.

### Treatment regimen

Both groups underwent chemotherapy according to the “Children’s Cancer and Leukemia Group-Acute Lymphoblastic Leukemia 2018 (CCLG-ALL2018)” protocol [10]. The initial treatment involved induction chemotherapy with Vincristine, Daunorubicin, L-asparaginase, and Prednisone (VDLP). Starting in May 2020, patients in the Combination Therapy Group received an additional cardioprotective agent (Dexrazoxane) alongside the VDLP induction chemotherapy. This agent was administered intravenously at a final concentration of 10 g/L over 30 min, with the dosage and total treatment cycles tailored to each patient’s individual condition. Following this, anthracycline chemotherapy was administered.

### Outcome measures

Cardiac Function and Damage Monitoring: Electrocardiogram (ECG) and Echocardiogram were utilized to assess cardiac structure and function, including any abnormal waveforms and the measurement of Left Ventricular Ejection Fraction (LVEF).

Biomarkers of Myocardial Injury: Cardiac Troponin T (cTnT), Creatine Kinase-Myocardial Band (CK-MB), and Myoglobin were measured in fasting venous blood samples using Enzyme-Linked Immunosorbent Assay (ELISA).

Adverse Reaction Monitoring: The incidence of all adverse reactions, including cardiotoxicity, has been documented.

### Statistical analysis

Statistical analyses were performed using SPSS software version 22.2. Categorical variables were expressed as percentages (%) and analyzed using Chi-square tests (χ² test). Continuous variables were presented as mean ± standard deviation (x̄ ± s) and compared between groups using independent samples t-tests or paired samples t-tests after testing for normality. A *P*-value of less than 0.05 was considered statistically significant.

## Result

### Baseline characteristics

Table 1 presents the baseline demographic and clinical characteristics of the study population. The two groups were well-balanced with respect to age (6.21 ± 1.12 vs. 6.35 ± 1.08 years, *P* = 0.315) and sex distribution (*P* = 0.896). Clinical characteristics including ALL risk stratification (*P* = 0.682), immunophenotype distribution (*P* > 0.999), initial white blood cell count (median 15.0 vs. 14.5 × 10⁹/L, *P* = 0.654), hemoglobin levels (10.5 ± 1.8 vs. 10.6 ± 1.7 g/dL, *P* = 0.782), platelet count (median 200 vs. 210 × 10⁹/L, *P* = 0.551), and CNS involvement (8.0% vs. 5.9%, *P* = 0.421) showed no significant differences between groups. Both groups received the CCLG-ALL2018 protocol with comparable cumulative anthracycline doses (120 ± 20 vs. 115 ± 18 mg/m², *P* = 0.320), confirming the comparability of the two cohorts.

**Table 1:** Baseline demographic and clinical characteristics of study population

Characteristics	Anthracycline Group (*n* = 125)	Combination Therapy Group (*n* = 135)	*P*-value
Demographics			
Age (years), mean ± SD	6.21 ± 1.12	6.35 ± 1.08	0.315
Sex, n (%)			0.896
Male	61 (48.8)	68 (50.4)	
Female	64 (51.2)	67 (49.6)	
Clinical Characteristics			0.682
ALL risk stratification, n (%)			
Standard risk	65 (52.0)	70 (51.9)	
Intermediate risk	45 (36.0)	48 (35.6)	
High risk	15 (12.0)	17 (12.6)	
Immunophenotype, n (%)			> 0.999
B-cell ALL	85 (68.0)	90 (66.7)	
T-cell ALL	40 (32.0)	45 (33.3)	
WBC count at diagnosis (*10^9^/L), median (IQR)	15.0 (8.0–30.0)	14.5 (7.5–28.5)	0.654
Hemoglobin (g/dL), mean ± SD	10.5 ± 1.8	10.6 ± 1.7	0.782
Platelet count (*10^9^/L), median (IQR)	200 (120–300)	210 (130–320)	0.551
CNS involvement, n (%)	10 (8.0)	8 (5.9)	0.421
Treatment Protocol			
CCLG-ALL2018 protocol, n (%)	125 (100)	135 (100)	1.000
Cumulative anthracycline dose (mg/m^2^), mean ± SD	120 ± 20	115 ± 18	0.320

Data are presented as mean ± SD, n (%), or median (IQR) as appropriate. *P*-values were calculated using independent t-test for continuous variables and chi-square test for categorical variables

*ALL* acute lymphoblastic leukemia, *WBC* white blood cell, *CNS *central nervous system, *CCLG* Children’s Cancer and Leukemia Group

### Comparison of incidence rates of electrocardiogram and echocardiogram abnormalities between groups

The incidence rates of abnormalities detected through ECG and echocardiogram assessments were significantly lower in the group receiving a combination of anthracycline and a cardioprotective strategy compared to the group treated with anthracycline alone. In terms of ECG abnormalities, the combination therapy group showed a significant decrease (2.22% vs. 12.00%, *P* = 0.0019). Table 2 provides detailed breakdown of specific ECG and echocardiographic abnormalities. The combination therapy group showed significantly lower total ECG abnormalities (2.22% vs. 12.00%, *P* = 0.0019) and total echocardiographic abnormalities (2.96% vs. 13.60%, *P* = 0.0019) compared to the anthracycline-only group.

**Table 2:** Detailed cardiac abnormalities detected by electrocardiogram and Echocardiogram after treatment

Cardiac Parameter	Anthracycline Group (*n* = 125)	Combination Therapy Group (*n* = 135)	Chi-square (χ²) Value	*P*-value
Electrocardiogram (ECG) Abnormalities				
QTc prolongation (> 450ms male/>470ms female)	10 (8.00%)	1 (0.74%)	7.81	0.005
First-degree atrioventricular block	3 (2.40%)	1 (0.74%)	1.09	0.296
Other arrhythmias (e.g., sinus tachycardia)	2 (1.60%)	1 (0.74%)	0.421	0.516
Total ECG Abnormalities	15 (12.00%)	3 (2.22%)	9.63	​0.0019
Echocardiogram (Echo) Abnormalities				
Mild valvular regurgitation	8 (6.40%)	2 (1.48%)	4.13	0.042
Tricuspid regurgitation gradient (TRG) > 30mmHg	4 (3.20%)	1 (0.74%)	2.07	0.150
Left ventricular shortening fraction (FS) < 28%	3 (2.40%)	1 (0.74%)	1.09	0.296
Longitudinal strain (LS) <−18%	2 (1.60%)	0 (0%)	2.02	0.155
Total Echo Abnormalities	17 (13.60%)	4 (2.96%)	9.63	0.0019

Data are presented as n (%)

*ECG* electrocardiogram, *Echo * echocardiogram, *QTc* corrected QT interval, *AV* atrioventricular, *TRG* tricuspid regurgitation gradient, *FS* fractional shortening, *LS* longitudinal strain

*P*-values were calculated using chi-square test or Fisher’s exact test as appropriate

### Comparison of Cardiac Markers Before and After Combination Therapy

There were no significant differences between the combination therapy group and the anthracycline-only group at baseline, according to the analysis of baseline and post-treatment cardiac markers, including LVEF, cTnT, Myoglobin, and CK-MB (all *P* > 0.05). Notably, compared to the anthracycline group, the combination therapy group showed a statistically significant improvement in cTnT levels after treatment (*P* < 0.05). While there were positive trends in the combination therapy group’s LVEF, Myoglobin, and CK-MB values, none of them reached statistical significance (all *P* > 0.05). Table 3 provides a detailed comparison of post-treatment cardiac biomarkers and functional parameters between the two groups.

**Table 3:** Post-treatment cardiac biomarkers and functional parameters

Indicator	Anthracycline Group (Mean ± SD) (*n* = 125)	Combination Therapy Group (Mean ± SD) (*n* = 135)​	T-value	*P*-value
Left ventricular ejection fraction (LVEF, %)	55.0 ± 3.0	55.1 ± 3.0	−1.15	0.253
Cardiac troponin T (cTnT, ng/mL)	0.070 ± 0.020	0.065 ± 0.020	2.48	0.014
Myoglobin (ng/mL)	73.0 ± 10.0	70.0 ± 9.0	1.88	0.062
Creatine kinase-MB (CK-MB, ng/mL)	0.65 ± 0.10	0.63 ± 0.09	0.94	0.347
QTc interval (ms)​	440 ± 25	430 ± 20	​−2.10	​0.037
Longitudinal strain (LS, %)​	−18.8 ± 2.5	−19.5 ± 2.0	2.30	0.022
Tricuspid regurgitation gradient (TRG, mmHg)	28 ± 6	25 ± 5	−3.20	0.001
FS (%)​	29 ± 3	30 ± 3	2.50	0.013

Data are presented as mean ± SD

*LVEF* left ventricular ejection fraction, *cTnT* cardiac troponin T, *CK-MB * creatine kinase-myocardial band, *QTc * corrected QT interval, *LS* longitudinal strain, *TRG* tricuspid regurgitation gradient, *FS* fractional shortening

P-values were calculated using independent t-test

### Comparison of adverse reaction incidence rates between treatment groups

The incidence rates of adverse events are assessed in this section for two patient cohorts: those treated with anthracycline alone and those treated with anthracycline plus a cardioprotective approach. Our statistical study disproved early ideas by finding no significant differences between the two groups in the incidence rates of important adverse reactions, such as rash, gastrointestinal problems, and impaired liver and kidney function (all *P* > 0.05). These results indicate that the risk profile for these particular adverse responses is not considerably changed by incorporating a cardioprotective strategy into anthracycline therapy. Table 4 presents a comprehensive comparison of adverse reaction incidence rates between the two treatment groups, with detailed breakdown of gastrointestinal reactions and other systemic adverse effects.

**Table 4:** Comparison of adverse reaction incidence between treatment groups

Adverse Reaction Category	Anthracycline Group (*n* = 125)	Combination Therapy Group (*n* = 135)​	χ^2^	*P*-value
Rash			0.458	0.499
Rash	5 (4.0)	9 (6.67)		
Gastrointestinal Reaction			0.171	0.679
Nausea	8 (6.4)	10 (7.41)		
Vomiting	6 (4.8)	7 (5.19)		
Diarrhea	4 (3.2)	3 (2.22)		
Abdominal Pain	3 (2.4)	2 (1.48)		
Stomatitis	2 (1.6)	1 (0.74)		
Abnormal Liver and Kidney Function			0.016	0.899
Elevated Liver Enzymes	5 (4.0)	6 (4.44)		
Jaundice (Liver-related)	1 (0.8)	2 (1.48)		
Abnormal Renal Function	1 (0.8)	1 (0.74)		
Total Incidence Rate			0.020	0.888
Total (at least one adverse reaction)	25 (20.0)	29 (21.48)		

Data are presented as n (%). Some patients experienced multiple adverse reactions; therefore, individual categories may sum to more than the total incidence rate. No severe (Grade 3–4) gastrointestinal complications such as bleeding or perforation were observed in either group

*P*-values were calculated using chi-square test

## Discussion

Anthracyclines, particularly daunorubicin, are cornerstone chemotherapy agents in the management of pediatric acute lymphoblastic leukemia, recognized for their significant antineoplastic effects. Despite their efficacy, the emergence of cardiotoxicity, manifesting in both clinical and subclinical forms, remains a major concern [11, 12].Research indicates that anthracycline therapy, especially at cumulative doses of 300 mg/m² or higher, is associated with a substantially increased risk of cardiotoxicity in cancer patients, with the risk escalating up to 23-fold, emphasizing the severity of this adverse effect [13, 14].The specific risk of cardiovascular complications from anthracycline-induced cardiotoxicity stands as a leading non-oncologic cause of morbidity and mortality in this demographic, potentially undermining the therapeutic successes against cancer.

The pediatric population treated with anthracyclines is particularly susceptible to cardiotoxicity, a vulnerability influenced by the developmental stage of their organ systems, including the cardiovascular system [15, 16].This emphasizes how critical it is to combine anthracycline therapy with efficient cardioprotective measures.

The introduction of cardioprotective strategies that interfere with mechanisms such as iron-mediated free radical formation and cell death caused by anthracyclines has been advocated to mitigate the incidence and severity of anthracycline-induced cardiotoxicity. This study demonstrated that the group receiving a combination of anthracycline and a cardioprotective strategy presented with significantly improved cardiac outcomes compared to the anthracycline-only group. This was demonstrated by increased cardiac troponin T levels after therapy and decreased incidence rates of abnormalities on electrocardiograms and echocardiograms [17, 18].The efficacy of these cardioprotective measures is well-documented, inhibiting lipid peroxidation, and thereby mitigating myocardial injury [19].Furthermore, the role of these strategies in enhancing the activity of hypoxia-inducible factor-1α (HIF-1α) contributes to their cardioprotective effects, aiding in the reduction of cardiac damage induced by anthracyclines [20].

Our results are consistent with previous research that highlights the important cardioprotective advantages of these strategies in pediatric oncology settings, especially when anthracycline therapies are being administered. The finding that the anthracycline-only group had elevated cardiac troponin levels underscores the drug’s early induction of myocardial injury and emphasizes the need for early intervention and surveillance in order to prevent anthracycline-induced cardiotoxicity [21].

Furthermore, a recent study discovered that the combination therapy group had better hematological parameters and a lower rate of adverse effects than the anthracycline-only group. These findings are consistent with the 2013 guidelines for managing cardiotoxicity associated with anthracycline therapy in cancer patients, which recommend the initiation of cardioprotective strategies from the first chemotherapy session and consistently applying them throughout the treatment course [22].

The mechanisms underlying these cardioprotective strategies, which may involve derivatives of ethylenediaminetetraacetic acid (EDTA) with high water solubility, are essentially based on the suppression of anthracycline-ion chelation. This inhibition significantly reduces the generation of free radicals, thereby lowering their concentration and mitigating myocardial cell damage caused by anthracyclines. Furthermore, the activation of HIF-1α through these strategies plays a crucial role in cardiac protection, as it enhances oxygen balance and promotes cell growth and recovery [23].

Recent research supports the effectiveness of cardioprotective strategies in reducing myocardial damage caused by anthracyclines in pediatric patients treated for ALL and other tumor-related conditions. A multicenter randomized controlled trial highlighted the significant cardioprotective role of these strategies in chemotherapy regimens involving anthracyclines [24].Given the dose-dependent cardiotoxicity of anthracyclines, the integration of cardioprotective strategies into treatment protocols emerges as a prudent approach to mitigate cardiac risks without compromising anti-tumor efficacy.

## Conclusion

In conclusion, our ambispective cohort study with historical controls demonstrates that dexrazoxane reduces certain markers of anthracycline-induced cardiotoxicity in pediatric ALL patients. Specifically, the combination therapy group showed significantly lower rates of ECG and echocardiographic abnormalities and improved cardiac troponin T levels, though changes in LVEF and CK-MB did not reach statistical significance. Importantly, the addition of dexrazoxane did not increase the overall incidence of non-cardiac adverse reactions (20.0%vs21.48%, *P* = 0.888). These findings suggest that dexrazoxane may offer cardioprotective benefits without additional toxicity in pediatric ALL patients receiving anthracycline therapy. However, larger prospective randomized trials are needed to confirm these findings and establish the long-term cardioprotective efficacy of dexrazoxane in this population.

## Data Availability

The datasets used and/or analysed during the current study are available from the corresponding author on reasonable request.

## Abbreviations

ALL: acute lymphoblastic leukemia
LVEF: left ventricular ejection fraction
ECG: Electrocardiogram
CK-MB: Creatine Kinase-Myocardial Band
VDLP: Vincristine, Daunorubicin, L-asparaginase, and Prednisone
